# Synaptic components are required for glioblastoma progression in *Drosophila*

**DOI:** 10.1371/journal.pgen.1010329

**Published:** 2022-07-25

**Authors:** María Losada-Pérez, Mamen Hernández García-Moreno, Irene García-Ricote, Sergio Casas-Tintó

**Affiliations:** 1 Instituto Cajal-CSIC, Madrid, Spain; 2 IIER-Instituto de Salud CarlosIII, Majadahonda, Spain; Harvard Medical School, Howard Hughes Medical Institute, UNITED STATES

## Abstract

Glioblastoma (GB) is the most aggressive, lethal and frequent primary brain tumor. It originates from glial cells and is characterized by rapid expansion through infiltration. GB cells interact with the microenvironment and healthy surrounding tissues, mostly neurons and vessels. GB cells project tumor microtubes (TMs) contact with neurons, and exchange signaling molecules related to Wingless/WNT, JNK, Insulin or Neuroligin-3 pathways. This cell to cell communication promotes GB expansion and neurodegeneration. Moreover, healthy neurons form glutamatergic functional synapses with GB cells which facilitate GB expansion and premature death in mouse GB xerograph models. Targeting signaling and synaptic components of GB progression may become a suitable strategy against glioblastoma. In a *Drosophila* GB model, we have determined the post-synaptic nature of GB cells with respect to neurons, and the contribution of post-synaptic genes expressed in GB cells to tumor progression. In addition, we document the presence of intratumoral synapses between GB cells, and the functional contribution of pre-synaptic genes to GB calcium dependent activity and expansion. Finally, we explore the relevance of synaptic genes in GB cells to the lifespan reduction caused by GB advance. Our results indicate that both presynaptic and postsynaptic proteins play a role in GB progression and lethality.

## Author summary

Glioblastoma (GB) is the most frequent and aggressive type of brain tumor. It is originated from glial cells that expand and proliferate very fast in the brain. GB cells infiltrate and establish cell to cell communication with healthy neurons. Currently there is no effective treatment for GB and these tumors result incurable with an average survival of 16 months after diagnosis. Here we used a *Drosophila melanogaster* model to search for genetic suppressors of GB progression. The results show that genes involved in the formation of synapses are required for glial cell number increase, expansion of tumoral volume and premature death. Among these synaptic genes we found that post-synaptic genes that contribute to Neuron-GB interaction which validate previous findings in human GB. Moreover, we found electro dense structures between GB cells that are compatible with synapses and that expression of pre-synaptic genes, including *brp*, *Lip-α* and *syt 1*, is required for GB progression and aggressiveness. These results suggest a contribution of synapses between GB cells to disease progression, named as intratumoral synapses.

## Introduction

Glioblastoma (GB) is the most lethal and aggressive tumor of the Central Nervous System. GB has an incidence of 3/100,000 adults per year [1], and accounts for 52% of all primary brain tumors. GB originates from glial cells or glial progenitors and causes death within 16 months after diagnosis [2] due to the low efficacy of standard treatments such as chemotherapy, radiotherapy or surgical resection.

In the last decade, *Drosophila melanogaster* has emerged as a reliable *in vivo* GB model that reproduces the features of human GB [3–9]. The GB condition is experimentally elicited by the expression of constitutively active forms of EGFR (Epidermal Growth Factor Receptor) and PI3K (Phosphoinositide 3-kinase) in glial cells, which are the two most common mutations in patients [9]. This experimental model has been previously used to study the contribution of RIO kinases [8], vesicle transport [6], the human kinase STK17A orthologue (Drak) [10,11], circadian rhythms [12] and several metabolic pathways in GB [13]. Consequently, the *Drosophila* model of GB is well characterized and suitable to study cellular properties of GB *in vivo*.

Tumor microenvironment and the communication between tumoral cells and neurons are crucial for GB progression and patient survival [3–5,14–17]. In addition, neuronal activity can also stimulate GB growth. Activity-dependent release of neuroliglin-3 (NLGN3) is required for GB progression in xenografts models, and NLGN3 induces the expression of synaptic proteins in glioma cells [18]. Moreover, GB samples show synaptic gene enrichment [19] and glioma cells form functional glutamate synapses with neighboring neurons, where GB cells are post-synaptic [18–20]. These studies also demonstrated that pharmacological or genetic inhibition of these electrical signals reduces growth and invasion of the tumor [19,20].

Synapses are the functional units which underlie animal behavior, memory and cognition. Chemical synapses are specialized asymmetric junctions between a presynaptic neuron and a postsynaptic target with different molecular composition, structure, and activities. Bruchpilot (Brp), Liprin alpha (Lip-α) and Synaptotagmin 1 (Syt 1) are conserved proteins localized in the presynaptic side.

Brp is a well-studied component of the presynaptic component of the synapses in *Drosophila* that accumulates in mature active zones (AZ). Brp is the orthologue of human AZ protein ELKS/CAST/ERC, and it is required for synapse formation [21]. Lip-α is a presynaptic scaffolding protein, orthologue to several human genes including PPFIA1 (PTPRF interacting protein alpha 1) and PPFIA2 (PTPRF interacting protein alpha 2). Lip-α directly interacts with tyrosine phosphatase receptors and it is involved in synapse formation, anterograde synaptic vesicle transport, neuron development, synapse organization and axon guidance [22–24]. Finally, Syt 1 is a pre-synaptic vesicle calcium binding protein that functions as the fast calcium sensor for neurotransmitter release at synapses [25].

Synapses elicit neurotransmission by mediating the clustering and fusion to the plasma membrane of neurotransmitters containing vesicles which release into the synaptic space [26]. The postsynaptic side is characterized by the accumulation of neurotransmitter receptors, including Glutamate receptors (GluR), the protein discs large (Dlg), orthologue of human PSD95 protein which mediates the clustering of postsynaptic molecules [27], and Synaptotagmin 4 (Syt 4), a vesicular calcium binding protein, directly implicated in retrograde signaling at synapses. Syt 4 is proposed to regulate calcium-dependent cargo trafficking within the postsynaptic compartment [28].

Benefitting from the conserved nature of most synaptic components, we set out to dissect the pre- versus post-synaptic contributions to GB progression using a *Drosophila* model of the human disease, in which the pathological condition of each cell type can be genetically manipulated. Thus, in addition to demonstrating that neuron-glioblastoma synaptogenesis is a conserved mechanism in GB progression, we show that synapse-like structures are also formed intratumoral and identify several synaptic genes required for GB expansion and premature death.

## Materials and methods

### Fly stocks

Flies were raised in standard fly food at 25°C, otherwise indicated.

Fly stocks used were *UAS-lacZ* (BL8529), *UAS-myr-RFP* (BL7119), *UAS-CD8GFP* (BL 5137), repo-Gal4 (BL7415), *tub-gal80ts* (BL7019), *elav-Gal4* (BL8760), *elav-lexA* (BL52676), *UAS-CD2*:*HRP* (BL8763), *UAS-Syt1-GFP* (BL6926), *lexAop-nSyb-spGFP1-10UAS-CD4-spGFP11* (BL64315), *UAS-nSyb-spGFP1-10lexAop-CD4-spGFP11* (BL64314), *UAS-mLexA-VP16-NFAT lexAop-rCD2-GFP* (CaLexA, BL66542), *UAS-Cameleon2*.*1* (BL 6901) *UAS-Syb^RNAi^ (BL38234), UAS-Liprin-alpha^RNAi^* (BL53868)*, UAS-Syt1^RNAi^* (BL31289)*, UAS-Syt4^RNAi^* (BL39016)*, UAS-Brp^RNAi^* (BL25891), *UAS-Brp^RNAi80449^* (BL80449, only for survival tests), *UAS-ShiTS* (BL 44222), Df(2)cl-h4 (BL6304), GluRIIA-GFP (BL23757), *TRE-RFP* (BL-59011), *UAS-Kir2*.*1* (BL 6595 and 6596), *UAS-nAChRα1 RNAi* (BL 28688), *UAS-nAChRα4 RNAi* (BL 31985), *UAS-KCNQ RNAi* (BL 27252) and *UAS-Caβ1 RNAi* (BL 29575) from the Bloomington Stock Center (https://bdsc.indiana.edu/index.html); *UAS-yellowRNAi* (KK106068), *UAS-GluRIIA-RNAi* (KK101686), *UAS-Dlg-RNAi* (KK109274) and *UAS-Bruchpilot-RNAi* (KK104630), *UAS-Shaker RNAi* (KK104474) and *UAS-ShakingB RNAi* (GD24578) from the Vienna Drosophila Resource Centre (https://stockcenter.vdrc.at/control/main); *UAS-dEGFRλ* and *UAS-dp110CAAX* gifted by R. Read; UAS-Liprinα-GFP [29], *GluRIIA-RFP* (Genomic fragment containing the GluRIIA gene including 1.2kb sequence upstream of the start codon and the sequence for red fluorescent protein (RFP) inserted in the C terminus after Ser893) [30] and Df^Δ22^ [31] gifted by S.J. Sigrist;*UAS-ihog-RFP* [32] gifted by I. Guerrero, *UAS-grnd Minos* gifted by P. Leopold, *C57-Gal4* gifted by Lori L. Wallrath, UAS-TNT gifted by Carolina Gómez Diaz.

The complete genotypes used in each experiment are listed in S1 Table.

### Inmunohistochemistry

Third-instar larval brains, were dissected in phosphate-buffered saline (PBS), fixed in 4% formaldehyde for 30 min, washed in PBS + 0.1 or 0.3% Triton X-100 (PBT), and blocked in PBT + 5% BSA for 1 hour. Samples were incubated overnight with primary antibodies diluted in block solution, washed in, incubated with secondary antibodies diluted in block solution for 2 hours and washed in PBT. Fluorescent labeled samples were mounted in Vectashield mounting medium with DAPI (Vector Laboratories).

Primary antibodies used were: mouse anti-Repo (DSHB 1:200), rabbit anti-GFP (Invitrogen A11122, 1:500), mouse anti-GFP (Invitrogen A11120, 1:500), mouse anti-Nc82(brp) (DSHB 1:30), rabbit anti-GluRIID (1:100) (gift from Dr. Stephan Sigrist, European Neuroscience Institute, Göttingen, Germany), mouse anti-ELAV (DSHB 1:50), Rabbit anti-Hrp (Jackson Immunoresearch 111-035-144, 1:400).

Secondary antibodies used were: anti-mouse Alexa 488, 568, 647, anti-rabbit Alexa 488, 568, 647 (Thermofisher, 1:500).

Images were acquired by confocal microscopy (LEICA TCS SP5).

### TEM

Transmission electron microscopy (TEM) was performed in CNS of 3rd instar larvae with horseradish peroxidase (HRP) genetically driven to glial cells (*repo-Gal4>UAS-HRP CD2*). Brains were fixed in 4% formaldehyde in PBS for 30 min at room temperature, and washed in PBS, followed by an amplification of HRP signal using the ABC kit (Vector Laboratories) at room temperature. After developing with DAB, brains were washed with PBS and fixed with 2% glutaraldehyde, 4% formaldehyde in PBS for 1h at room temperature. After washing in a phosphate buffer the samples were postfixed with OsO4 1% in 0.1 M 7phosphate buffer, 1% K3[Fe(CN)6] 1h at 4°C. After washing in dH2O, Brains were incubated with tannic acid in PBS for 1 min at room temperature then washed in PBS for 5min and dH2O 2x5min. Then the samples were stained with 2% uranyl acetate in H2O for 1h at room temperature in darkness followed by 3 washes in H2O2d. Brains dehydrated in ethanol series (30%, 50%, 70%, 95%, 3x100% 10 min each at 4°C). Infiltration: samples were incubated in EtOH:propylene’s OXID (1:1;V.V) for 5 min, propylene’s OXID 2x10min, propylene’s OXID:Epon (1:1) for 45 min, Epon 100% in agitation for 1 h and Epon 100% in agitation overnight. Then change to Epon 100% for 2–3 h. After, the samples were encapsulated in BEEM and incubated 48h at 60°C for polymerization. Finally, the samples were cut in ultra-fine slices for TEM imaging [33].

### Imaging

Fluorescent images were acquired by confocal microscopy (LEICA TCS SP5) and were processed using Fiji (Image J 1.50e). These images were quantified with Fiji (Image J 1.50e) or Imaris 6.3.1 (Bitplane) software. The images of the ultra-fine slices were taken with a Transmission electron microscopy JEM1010 (Jeol) with a CMOS TemCam F416 (TVIPS) camera and processed with Adobe Photoshop CS4. Figures were assembled using Adobe Photoshop CS4 and Adobe Illustrator CS4.

IMARIS quantification (Imaris 6.3.1 software): The number of glial cells (Repo+) and the number of synaptic active sites was quantified by using the spots tool. The tumor volume was quantified using the surface tool. We selected a minimum size and threshold for the puncta or surface in the control samples of each experiment to establish the conditions. Then we applied the same conditions to the analysis of each corresponding experimental sample.

Fiji quantification:

- CaLexA expression: We used the NFAT-based neural tracing method-CaLexA (calcium-dependent nuclear import of LexA)-for labeling active neurons in behaving animals to measure calcium activity in glial cells. CaLexA (green) signal intensity was determined using ImageJ to calculate the mean gray value of each brain lobe.
- Cameleon expression: We used the genetically encoded calcium-sensitive fluorescence protein Cameleon 2.1. Signal intensity was determined using ImageJ to calculate the mean gray value of four different regions in each brain lobe
- Syt1-GFP expression: Syt1-GFP (green) signal and glia membrane myrRFP (red) signal intensity were determined using ImageJ (mean gray value) in three single slices at the middle of each brain lobe to calculate the ratio GFP/RFP.

All samples were treated, acquired and measured under the same conditions and in parallel

- GRASP: We used a modified version of this system to specifically detect synaptic contacts. It is based on the fusion of synaptobrevin protein (Syb) to the 1–10 fragment of GFP (Syb-GFP_1-10_), and the expression of a membrane bound form of the 11 fragment of GFP (CD4-GFP_11_). UAS-nSyb*-spGFP^1-10^*:lexAop-*CD4-sp GFP^11^* (BL62314) and lexAop-*nSyb-spGFP^1-10^*: UAS-*CD4-sp GFP^11^* (BL62315) were expressed in neurons (*elav-lexA*) and glial (*repo-Gal4*) cells respectively. These complementary GFP fragments will reconstitute a functional fluorescent reporter at the points of contact and therefore, it will allow the identification of the presynaptic and postsynaptic cells (e.g. glia and neuron) [34].

### Viability assays

Flies were crossed at restricted temperature (17°C, to inactivate the *UAS/Gal4* system with *tub-Gal80ts*) for 4 days then progeny was transfer at 29°C (when the *UAS/Gal4* system is active and the glioblastoma develops). The number of adult flies emerged from the pupae were counted for each genotype. The number of control flies was considered 100% viability and all genotypes are represented relative to controls. Experiments were performed in triplicates.

### Survival assay

For survival analyses of adult flies, males and females were analyzed separately. 0–5 day old adult flies raised at restricted temperature were put at 29°C in groups of 10 animals per vial and were monitored blinded every 2–3 days; each experiment was done at least three times.

### Quantifications and statistical analysis

All experiments including different genotypes were done in parallel under the same experimental conditions, with the exception of viability analysis where each genotype was normalized with their parallel control. Data were analysed and plotted using GraphPad Prism v7.0.0 and Excel (viability assays). A D’Agostino & Pearson normality tests were performed and data with normal distributions were analysed using a two-tailed T-test with Welch-correction. If data had multiple comparisons, a One-way ANOVA with Bonferroni posthoc-test was used. Data that did not pass normality testing were submitted to a two-tailed Mann-Whitney U-test or where the data had multiple comparisons a Kruskal-Wallis test and Dunnett’s post hoc-test. Error bars represent Standard Error of the Mean, significance values are: ***p≤0.0001, ** p≤0.001, *p≤0.005, *ns* = non-significant.

## Results

**Fig 1:**
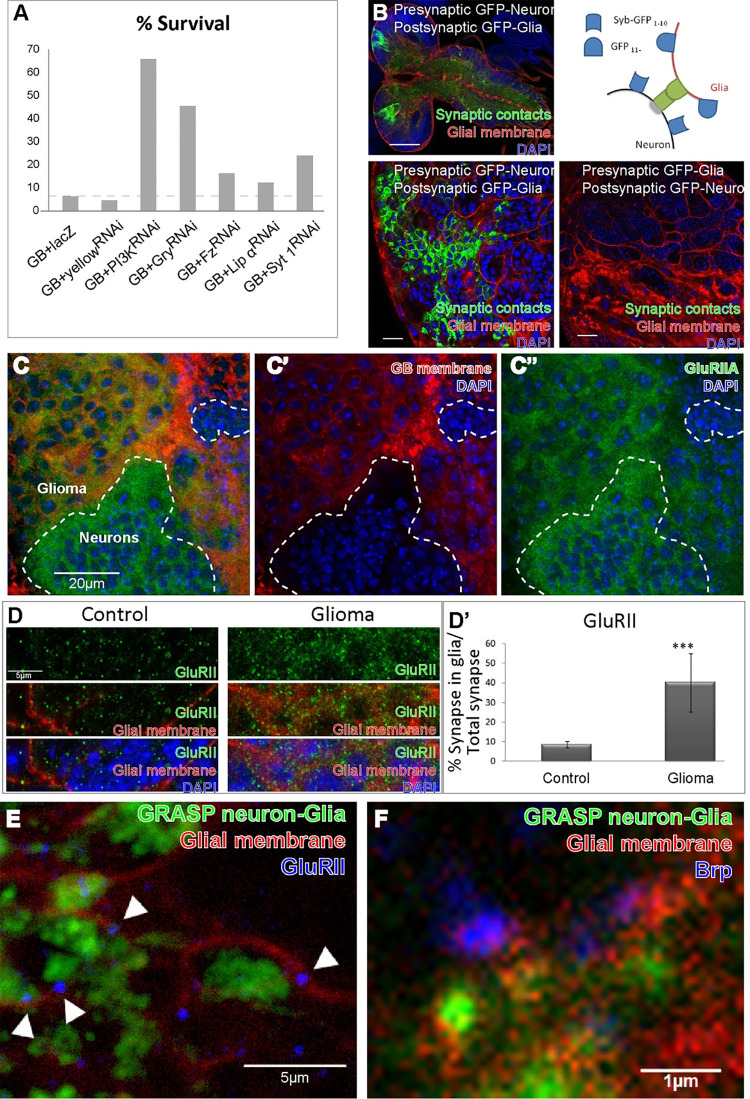
GB cells form synapses with neurons. A) Histogram showing the percentage of viability of flies when glioblastoma (GB) is induced alone (GB+lacZ or GB+yellowRNAi) or combined with *PI3K*, *gry*, *fz*, *lip α* or *syt 1* knockdown in GB cells by RNAi. Percentage corresponds to the number of adult flies that emerged from the pupae for each genotype, relative to the controls (siblings without *repoGal4*, considered 100% of viability). Absolute numbers and percentages are in S1 Table, B top left) confocal image of GRASP+ signal in larval brain when presynaptic Syb-GFP_1-10_ fragment is expressed in neurons and CD4-GFP_11_ fragment is expressed in GB cells; B bottom left) magnification of above; B top right) Diagram of GRASP technique; B bottom right) confocal image of GRASP–signal when presynaptic Syb-GFP_1-10_ fragment is expressed in glioma cells and CD4-GFP_11_ fragment is expressed in neurons; Synaptic contracts are shown in green (GFP), glial membrane are in red (repo>myrRFP) and all nuclei (DAPI) are in blue. C) Confocal image of larval GB brain, carrying the protein-trap GluRIIA-RFP (green), showing glial membrane (repo>GFP, red) surrounding healthy tissue (neurons, not red). Dotted lines mark the limit between neurons and glioma cells. GluRIIA postsynaptic protein is detected in both GB and healthy tissue (C and C”). DAPI staining nuclei is in blue. D) GluRIID staining (green) in control and GB larval brains, presented with glial membrane or glial membrane and DAPI in the bottom images. D’) Number of GluRIID-positive dots overlapping with glial membranes in control and GB (No. = 6 brain lobes) samples. Statistic: Unpaired T-Test (* p<0.05). E) High magnification confocal image showing GB membrane (red) and Neuron-Glia GRASP signal (green) in the proximity of Brp signal (blue). F) High magnification confocal image showing GB membrane (red) and Neuron-Glia GRASP signal (green) in the proximity of GluRIID signal (blue). Scale bars: 100μm (B up), 10μm (B down), 20μm (C), 5μm (D and E) and 1μm (F). Raw numbers and complete genotypes are in S1 Table.

We performed a *Drosophila* biased genetic screening to search for relevant genes related to GB progression. We selected 2000 genes involved in cell to cell communication, and we used VDRC UAS-RNAi lines to knockdown the expression of such genes encoding transmembrane, secreted and cell to cell communication proteins. In addition, we used the previously validated EGFR/PI3K model [5,6,9,16]. GB induction in larvae causes premature death and animals do not reach adulthood. We took advantage of this unequivocal phenotype as a read-out, quantifying the number of adult flies that emerged from each experiment. We obtained 25 RNAi lines that rescued the lethality caused by the GB. Among the suppressors, we found well known mediators of GB progression such as *Frizzled1* (*Fz1*) or *Gryzun (Gry)* and PI3K signaling pathway members [5,6,9]. These genes validate the experiment as positive controls. Most RNAi lines, as well as negative controls UAS-*yellow* RNAi or UAS-*beta-galactosidase (lacZ)*, did not rescue GB-induced pupal lethality however, we found RNAi lines against synaptic genes, such as *Liprin-α (Lip-α) and synaptotagmin1 (syt 1)* that rescue GB-induced lethality (Fig 1A). These results motivated this study to determine the contribution of synaptic components to GB progression.

### Neurons produce synaptic contacts with glioma cells

It was recently described that neurons establish functional synapses with glioblastoma cells in mouse xenografts [19,20]. In these studies, GB cells are postsynaptic with respect to neurons, however our results from the screening indicate that presynaptic genes are also involved in GB-induced lethality (Fig 1A). Our previous results suggested that neurons and GB cells establish an intimate contact with neurons, compatible with synaptic distance [5]. Therefore, we wondered if GB cells were pre- or postsynaptic in the *Drosophila* GB model. We used a modified version of the GFP reconstitution across synaptic partners (GRASP) technique [35] to determine synaptic contacts between GB cells and neurons. This technique allows the identification of pre- and postsynaptic cells (see Materials and Methods). The confocal images of larvae brains show that GFP signal is reconstituted (GRASP+) if presynaptic Syb-GFP_1-10_ fragment is expressed in neurons, under the control of the specific neuronal enhancer *elav-lexA*, and CD4-GFP_11_ fragment is expressed in GB cells under the control of the specific glial enhancer *repo-Gal4* [36] (Fig 1B). In addition, we co-expressed a myristoylated form of Red Fluorescent Protein (myrRFP) in glial cells under the control of the UAS/Gal4 system to visualize GB cells membranes. In contrast, GFP does not reconstitute when GB cells express the presynaptic component of GRASP, and neurons express the post-synaptic component (Fig 1B). These results indicate that neurons (pre-synaptic) establish synapses with GB cells (post-synaptic) in *Drosophila*. These contacts occur in a unidirectional manner, therefore validating previous results in other GB model systems.

Next, to further explore the postsynaptic role of GB cells, we studied the expression of the post-synaptic *Glutamate receptor II* (*GluRII*) gene in GB cells. We used the GluRIIA-RFP protein trap transgenic line to monitor the expression and localization of GluRIIA protein [29] (S1A–S1A” Fig). Confocal images (Fig 1C–1C”) show GB tissue (red) and not-tumoral healthy tissue (not-red), and we observed the presence of GluRIIA-RFP signal in GB tissue (Fig 1C”). Moreover, to confirm the presence of GluRII protein in the membranes of GB cells, we used a validated antibody against the GluRIID subunit. Confocal images of control larval brain samples showed GluRIID dotted signals through the brain revealing glutamatergic synapses (Fig 1D). We used confocal images from control larvae brains and GB brains, and quantified with IMARIS the total number of GluRIID dots, and the number of GluRIID positive dots that overlap with glial membranes (mRFP). We aimed to compare the green signal of GluRIID antibody that overlaps with the red signal from the glial membrane. In the glioma samples, the images and quantifications show that green signal overlapping with red signal increases. In the image corresponding to glioma, the green pattern reproduces the pattern of glial membrane, and we did not observe an increase of GluRIID signal out of glial membrane. We have considered the total GluRIID signal to make the ratio shown in Fig 1D´. The results show that less than 10% of the total GluRIID signal corresponds to glial membranes in control samples, whereas the number of GluRIID positive signals in the GB membrane reaches 40% of total synapses (Fig 1D and 1D’). In summary, our data suggest that GluRIID protein accumulates in GB cells.

To further determine the nature of these synaptic structures, we co-stained GB larval samples with a specific monoclonal antibody that recognizes the pre-synaptic protein Bruchpilot (Brp), or an antibody against GluRIID and analyzed the relative position with GRASP signal. High magnification confocal images show GB membrane (red) and Neuron-Glia GRASP signal (green) in the proximity of Brp signal (blue in Fig 1E) or GluRIID signal (blue in Fig 1F). In both cases, proteins appear at less than 1 micrometer distance (Fig 1E and 1F) compatible with the formation of synapses. These results suggest that pre- and post-synaptic proteins accumulate in the GB-neuron contact region.

### GluRIIA and dlg post-synaptic proteins are required for GB expansion

**Fig 2:**
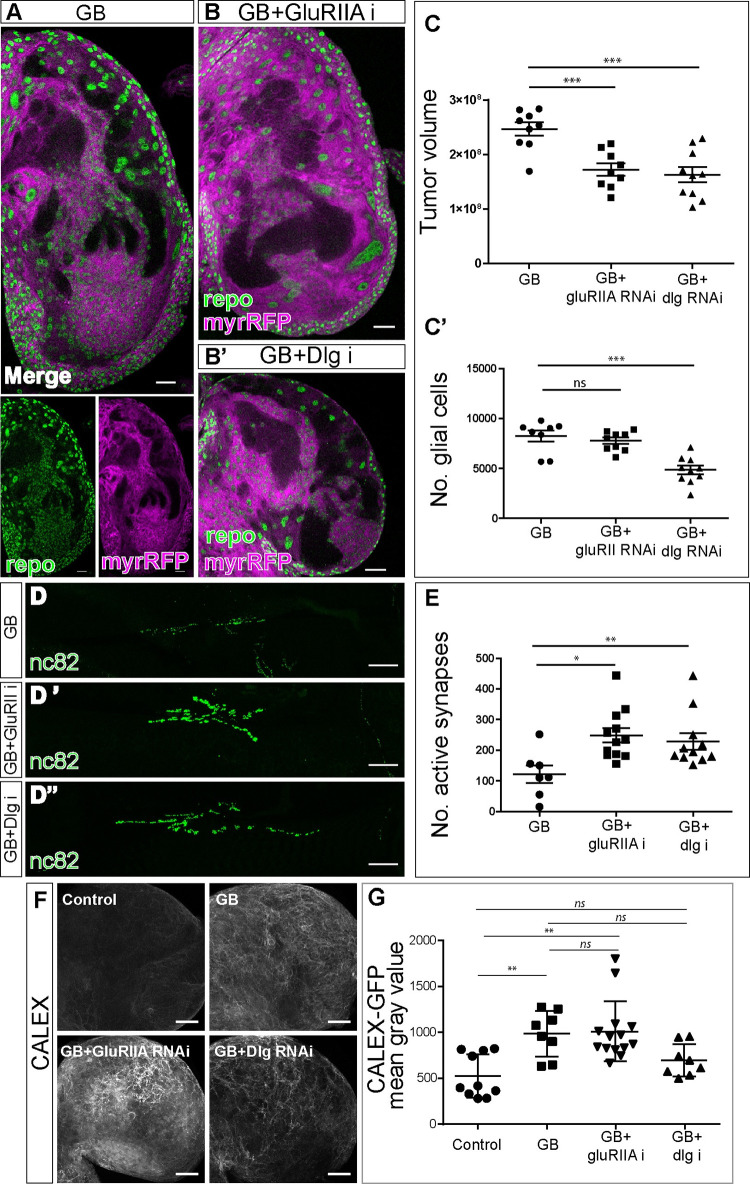
Postsynaptic proteins GluRII and Dlg are required for GB progression. A) Representative confocal image of GB larval brain lobe showing glial nuclei (green) and glial membrane (magenta). Bottom images show green and magenta channels separately. B-B’) Representative confocal images of GB larval brain lobe + *GluRIIA RNAi* expression (B) or *dlg RNAi* expression (B’) in GB cells. Glial nuclei are shown in green and glial membranes in magenta. C) Quantification of GB membrane volume (C, No.≥ 8 brain lobes) or number of glial cells (C’, No.≥ 8 brain lobes) in GB and GB with *GluRIIA* or *dlg* RNAi. Statistics: Dunnett’s Multiple Comparison Test (***p<0.0001). D) Confocal images of Neuromuscular junctions showing Brp (green) accumulation in GB (D) and GB with *GluRIIA RNAi* (D’) or *dlg* RNAi (D”). E) Quantification of synapse number (Brp positive dots) in GB and GB with *GluRIIA RNAi* or *dlg RNAi*. No.≥ 7 NMJs. Statistics: Dunnett’s Multiple Comparison Test (** p<0.001, *p<0.005). F-G) Representative confocal images of larval brain lobes showing CALEX signal (F) and quantifications (G) of CALEX mean gray value in control brains, GB brains and GB upon *dlg* or *GluRIIA* knockdown. No.≥ 8 brain lobes. Statistics: Dunnett’s Multiple Comparison Test (** p<0.001, *ns* = non-significant). Scale bars: 50 μm (A, B, D-D” and F). Raw numbers and complete genotypes are in S1 Table.

Once we have demonstrated the postsynaptic nature of GB cells we aimed to determine their contribution to GB progression. Third instar larvae brains with GB display expanded glial membrane, formation of perineuronal nests [5] (magenta in Fig 2A) and a subsequent increase of brain volume (Fig 2A). We used validated RNAi lines to knockdown *dlg* or *GluRII* postsynaptic genes in GB cells (GluRIIA-RNAi validated in S1A–S1A” Fig; GluRIIA-RNAi and dlg-RNAi validated in S1B Fig). The quantifications of the results show that *dlg* or *GluRII RNAi* prevents membrane expansion, disrupts perineuronal nest formation and prevents brain size increase (Fig 2B, 2B’ and 2C).

Next, we analyzed the contribution of *dlg* and *GluRII* to the increase of glial cell number in GB, we stained brain samples with the specific anti-repo antibody to visualize glial nuclei (Fig 2B and 2B’). The quantifications show that *dlg* knockdown reduces significantly the number of GB cells, but, on the contrary, *GluRIIA RNAi* expression does not prevent the increase in glial cell number in GB (Fig 2C’). To further validate the GluRIIA-RNAi tool we analyzed the number of cells and tumor volume in GB combined with GluR somatic mutants (S1C and S1C’ Fig). These results showed that both heterozygote and trans-heterozygous *Df(2)clh4/Df(GluRIIa-GluRIIb-)^Δ22^* GluR mutants reduce cell number and tumor volume.

Given that downregulation of *GluRII* or *dlg* prevent the GB membrane expansion (Fig 2C) and that the expansion of tumor microtubes in GB cells mediates the reduction of synapses in neighboring healthy neurons [3,5,6,15], we wondered if *GluRII* and *dlg* were required for the GB-induced synapse number reduction. We analyzed larval neuromuscular junctions (NMJ) which is a validated system to count synapse number [37,38]. Additionally, we have seen in previous work that synapse number reduction at NMJ correlates with GB progression [3,5]. We quantified the number of synapses by counting the number of active zones (Brp positive dots, green in Fig 2D–2D”). The results indicate that GB progression causes a reduction of synapse number, as previously reported, and that *GluRII* or *dlg* knockdown in GB cells prevents this reduction (Fig 2E). Thus, the expression of *dlg* and *GluRII* post-synaptic genes in GB cells is required for the reduction in synapse number in NMJs.

Cell proliferation in GB is associated with calcium-mediated activity [20], thus we analyzed the contribution of *dlg* or *GluRIIA* to calcium activity in GB cells. To monitor calcium activity, we used the CaLexA system (see Materials and Methods). Confocal images and quantification of CaLexA signal showed a significant increase of calcium signal in GB samples (Fig 2F and 2G). To further confirm the increase of calcium signal in GB we used Cameleon, an additional reporter of calcium activity [39,40], and found similar results (S1D–S1D” Fig). CaLexA signal in GB upon *dlg* knockdown maintained calcium levels as controls (Fig 2F and 2G). However, we did not find significant differences upon *GluRIIA* downregulation. These results indicate that GB cells show enhanced calcium-dependent activity, in line with previous data from other GB models [19]. Moreover, our data indicate that this enhanced calcium activity is dependent on *dlg* expression, while independent on that of *GluRIIA*.

### Vesicle calcium binding proteins are required for GB progression

Vesicle calcium binding, vesicle transport and neurotransmitter release are cellular mechanisms related to synaptic function [41,42]. We have found that GB has an enhanced calcium activity that can be reduced by downregulating the expression of *dlg*. Moreover, our screening results indicate that downregulation of *syt 1*, which encodes a presynaptic Calcium-binding protein, partially rescued the lethality caused by the GB (Fig 1A). This motivated the study of Synaptotagmin 1 (Syt 1), as well as Synaptotagmin 4 (Syt 4, a postsynaptic Ca-binding protein) in GB progression.

**Fig 3:**
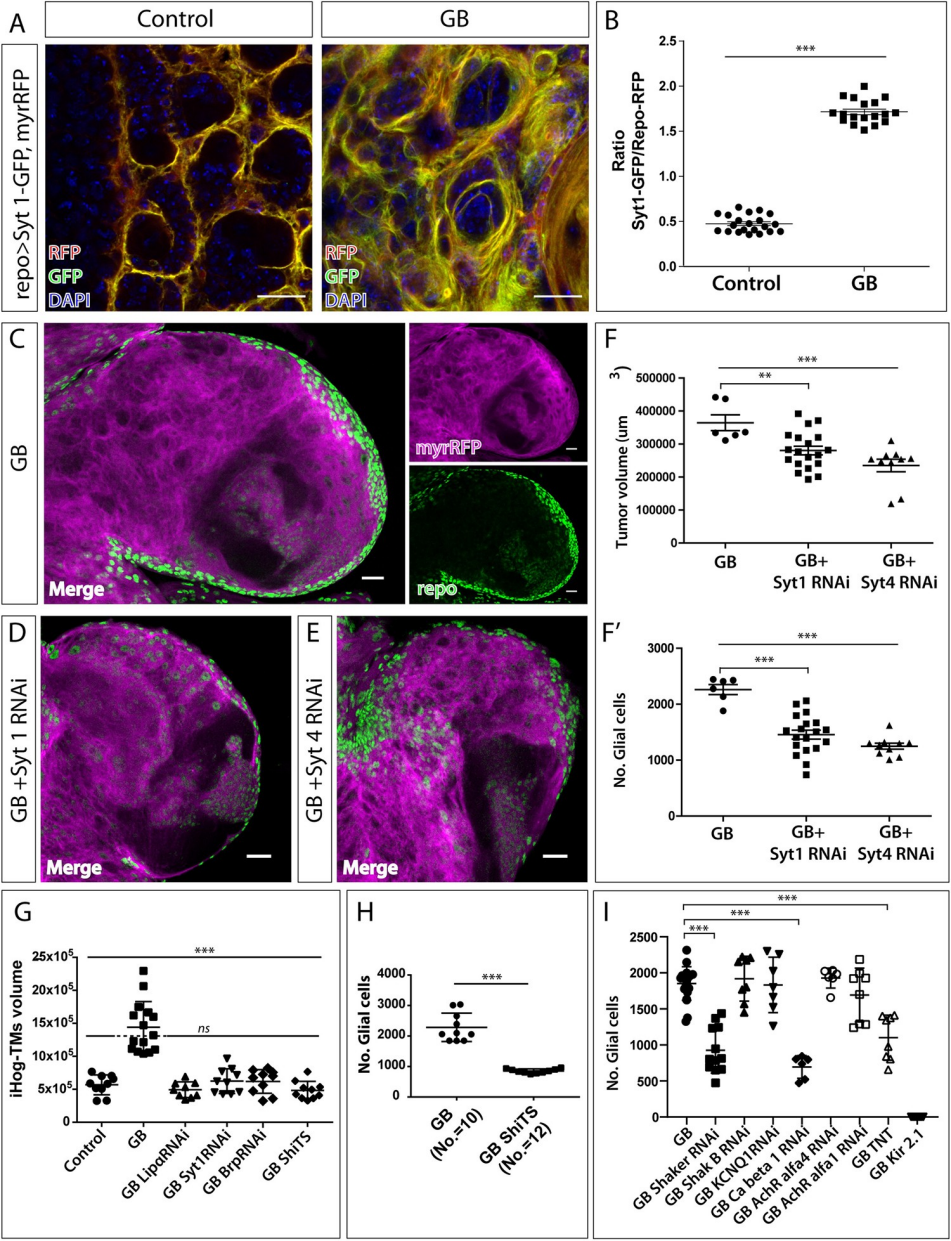
Sygnaptotagmin 1 and 4 are required for GB progression. A) Representative confocal images showing Syt 1-GFP accumulation (green) in control (left) and GB brains (right). Glial membrane is shown in red and nuclei (DAPI) in blue. B) Quantification of GFP/RFP ratio, corresponding to Syt1-GFP/glial membrane in control and GB brains. No.≥ 18 brain lobes. Statistics: Unpaired T-Test with Welch’s correction (***p<0.0001). C) Representative confocal image of GB larval brain lobe showing glial nuclei (green) and glial membrane (magenta). Right images show green and magenta channels separately. D-E) Representative confocal images of GB larval brain lobe expressing *Syt 1 RNAi* (D) or *Syt 4 RNAi* (E). Glial nuclei are shown in green and glial membranes in magenta. F) Quantification of glial membrane volume (F) or number of glial cells (F’) in GBl and GB + *Syt 1* or *Syt 4* knockdown. No.≥ 6 brain lobes. Statistics: Dunnett’s Multiple Comparison Test (***p<0.0001, ** p<0.001,). G) Quantification of TMs volume (RFP signal of repo>UAS-ihog-mRFP) in controls, GB, GB with presynaptic genes (syt1, Lip-alpha or brp) downregulated or GB combined with the dynamin temperature sensitive allele Shibire (*shiTS*). No.≥ 9 brain lobes. Statistics: Dunnett’s Multiple Comparison Test (***p<0.0001, *ns* = non-significant. H) Quantification of the number of glial cells in larval brains in GB and GB combined with *shiTS* in glial cells. No. = 10 brain lobes. Statistics: T-Test (***p<0.001). I) Quantification of the number of glial cells in GB and GB combined with the expression of shaker-RNAi, shakingB-RNAi, KCNQ1-RNAi, calcium beta1 subunit-RNAi, Acetylcholine alfa 4-RNAi, Acetylcholine alfa 1-RNAi, Kir2.1 or Tetanus toxin, TNT. No.≥ 6 brain lobes. Statistics: Dunnett’s Multiple Comparison Test Test (***p<0.0001). Scale bars: 15 μm (A) and 50 μm (C-E). Raw numbers and complete genotypes are in S1 Table.

To explore the pre-synaptic role of GB, we analyzed Syt 1 accumulation in normal glia and GB cells. We used a Syt1-GFP fusion protein to label synaptic vesicles and measure the accumulation of Syt 1 in glial membranes, comparing normal glia with GB cells. The confocal images show that Syt 1 accumulates in the membrane of glial cells, and this accumulation exacerbates in GB samples (Fig 3A and 3B).

We used specific RNAi tools to knockdown *syt 1* or *syt 4* expression (RNAi lines validated for disrupting synapses when expressed in neurons (*elav-Gal4*) [36] *or* muscle respectively (*C57-Gal4*) [43] (S1B Fig), and studied the effects on glial cell number and GB volume. The quantifications showed that *syt 1* or *syt 4* knockdown specifically in GB cells prevents the expansion of GB and reduces the number of GB cells (Fig 3C–3F’). Therefore, the expression of these two genes that regulate vesicle transport and neurotransmitter release are required for GB development in *Drosophila*. These results support the post-synaptic nature of GB cells (*syt 4*), and also support a pre-synaptic condition (*syt* 1) of GB cells as a requirement for GB expansion.

Recent reports showed that components of neuronal synapses function in proper cytoneme formation and signaling in the development of epithelial tissues in *Drosophila* [44]. GB cells expand a network of specialized cytonemes named Tumor Microtubes (TMs) [5,45]. To determine if TMs formation in GB cells depends on the expression of presynaptic genes, we knocked down the expression of *syt 1*, *lip-α* or *brp* in GB cells (*lip-α* and *brp RNAi* lines also reduce synapse when expressed in neurons. S1B Fig). To visualize TMs, we co-expressed an RFP tagged form of *ihog* (*ihogRFP*) previously validated that marks cytonemes and TMs [5,32] (Fig 3G). The results indicate that *syt 1*, *lip-α* or *brp* expression in GB cells is also required for TMs growth. Given the similarities between cytonemes and TMs in GB, we conclude that cytonemes and TMs share similar molecular mechanisms.

Additionally, to determine if synaptic activity is required for GB progression and TMs expansion, we analyzed the number of GB cells and the volume of TMs in flies expressing a thermosensitive dynein dominant negative allele *Shibire TS* (*ShiTS*) in GB. This mutant form blocks the endocytosis of synaptic vesicles in synaptic boutons at non permissive temperatures [46].

The quantifications show that expression of *ShiTS* in GB cells also rescues TMs volume (Fig 3G) and GB cells number (Fig 3H). These results suggest that functional synapses are required for GB development.

Next, to determine if GB progression depends on the contribution of synaptic activity as a general mechanism, we modified one by one the expression of specific genes encoding ionic channels in GB cells or directly associated with synaptic activity, and quantified the number of GB cells in *Drosophila* larvae brains. To this aim, we used RNAi against *shaker*, the structural alpha subunit of a voltage-gated potassium channel [47,48], *shakingB*, a structural component of the gap junctions at electrical synapses [49–51], *KCNQ1*, a voltage-gated potassium channel [52,53], *calcium beta1* subunit, a voltage-gated calcium channel [54] or *Acetylcholine alfa 4 or alfa 1 receptor subunits* [55,56]. Besides, we overexpressed *Kir2*.*1* (gene KCNJ2), a rectifying potassium channel that allows more potassium ions to enter the cell [57,58], or *tetanous toxin* (UAS-TNT) in GB cells [59,60] (Fig 3I). All these strategies are directed towards the disruption of synaptic activity in GB cells. The results show that *shaker* or *Ca beta1 RNAi* and *TNT* overexpression in GB cells, prevent GB progression. Additionally, *Kir2*.*1* overexpression in GB cells resulted in embryonic lethality therefore, we cannot have a clear conclusion on the contribution of potassium-dependent signals in GB progression and viability. On the contrary, no further genetic combinations showed a significant change in GB cells number, indicating that synaptic activity as a general mechanism is not involved in GB progression. These results suggest that calcium and potassium signaling contribute to the expansion of GB cell number, but the specific involvement of potassium signals to GB progression will require further investigation.

### Presynaptic proteins are required for the enhanced GB calcium activity

**Fig 4:**
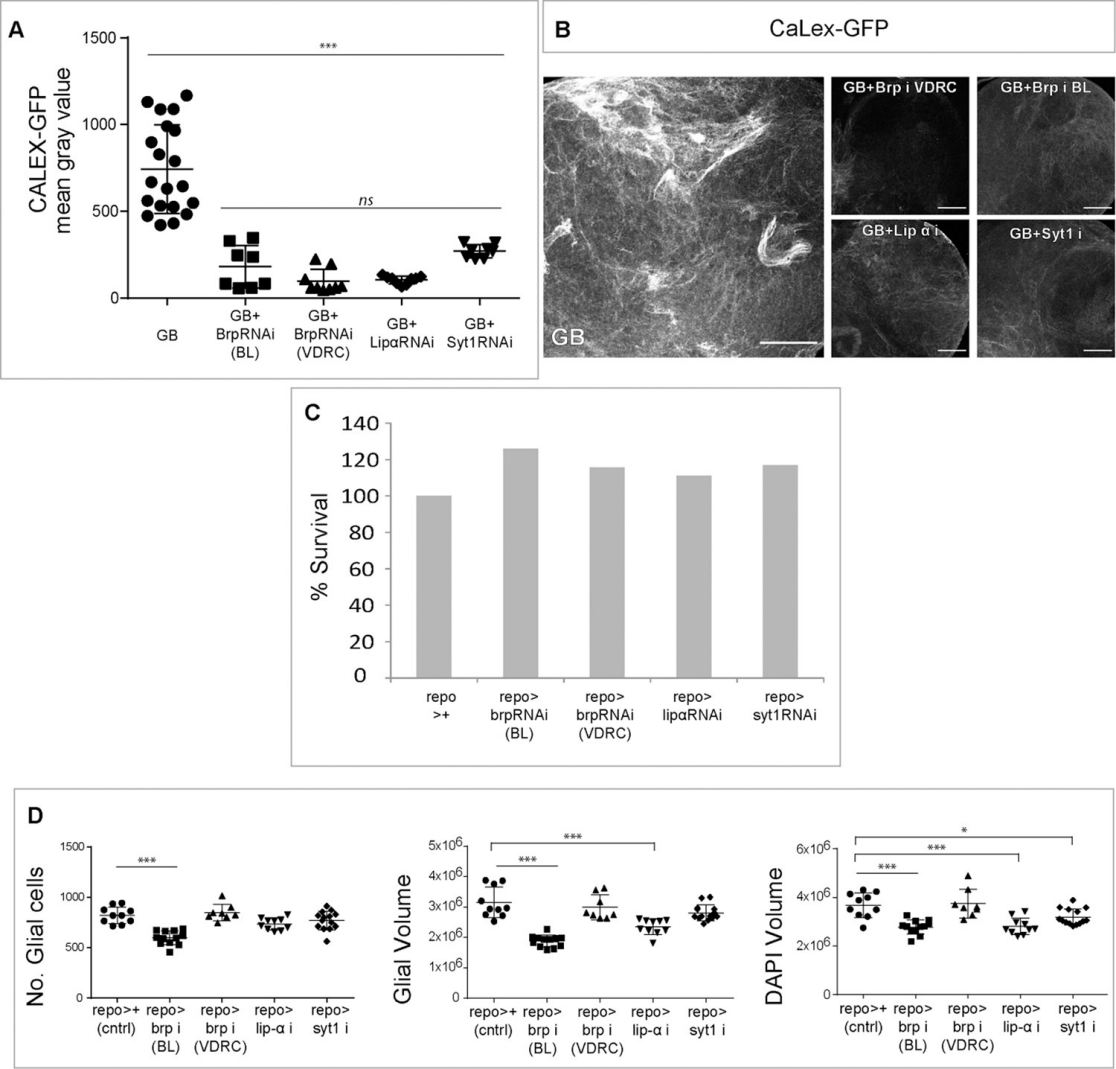
Presynaptic proteins are required for Calcium influx in GB. A) Quantifications of CaLexA signal in GB brains, and GB brains upon presynaptic genes *brp*, *syt 1* or *lip-ɑ* knockdown by RNAi. No.≥ 8 brain lobes. Statistics: Bonferroni’s Multiple Comparison Test (***p<0.0001, *ns* = non-significant). B) Representative confocal images of CaLex signal in GB brains, and GB brains upon presynaptic genes *brp*, *syt 1* or *lip-ɑ* knockdown by RNAi. C) Histogram showing the viability of control flies (100%) compared with flies where *brp* (134.26% and 108.5%), *lip* (121.26%) *α* or *syt 1* (103.19%) are downregulated in normal glia. No.≥ 106 animals. D) Quantification of the number of glial cells, glial membrane volume and DAPI volume, per larval brain lobe in controls, and animals where *brp*, *lip α* or *Syt 1* are downregulated in glia. No.≥ 8 brain lobes. Statistics: Dunnett’s Multiple Comparison Test (***p<0.0001, *p<0.005). Scale bars: 50 μm. Raw numbers and complete genotypes are in S1 Table.

To further explore the presynaptic condition of GB cells, we measured the accumulation of intracellular calcium by knocking down the presynaptic genes *brp*, *syt 1* or *lip-α* in GB cells. The confocal images as well as the quantification of CaLexA intensity signal showed that the knockdown of these presynaptic genes prevented the increase of CaLexA signal in GB cells (Fig 4A and 4B). This suggests that presynaptic genes are required for the induction of calcium accumulation and calcium-dependent activity in GB cells.

To ensure that presynaptic proteins have a specific role in GB progression and are not required for normal glia development we analyzed the viability of animals where *lip-α*, *brp* or *syt 1* are knocked down in glial cells (through all developmental stages under the control of *repo-Gal4)*. The quantification shows that, in all cases, the percentage of animals that reach adulthood compared with controls (the siblings) is similar or even higher (Fig 4C), and therefore, we conclude that downregulation of *lip-α*, *brp* or *syt 1* in glial cells does not affect viability and therefore, are not required for vital functions during development.

Additionally, we dissected brains of third instar larvae and quantified the number of glial cells, the volume of glial membrane and brain size (Fig 4D). The results show that the number of glial cells was normal in all cases with the exception of the expression of *Brp RNAi BL*. In addition, we measured the total volume of glial cells membrane marked with myrRFP. The quantification of RFP volume indicates that the expression of *Brp RNAi VDRC* and *syt 1 RNAi* did not cause any effect during development; however, *Brp RNAi BL* and *Lip-α RNAi* expression in glial cells caused a reduction of the total volume of glial cells membranes (Fig 4D). Finally, we measured the total brain volume marked with DAPI, brain volume was reduced upon *Brp BL RNAi*, *Lip-α RNAi* and *syt 1 RNAi* expression, but not upon *Brp RNAi VDRC* expression (Fig 4D). These data unveil a role of these synaptic proteins in the biology of normal glial cells that was unknown hereto.

### Intratumoral synapses in GB

**Fig 5:**
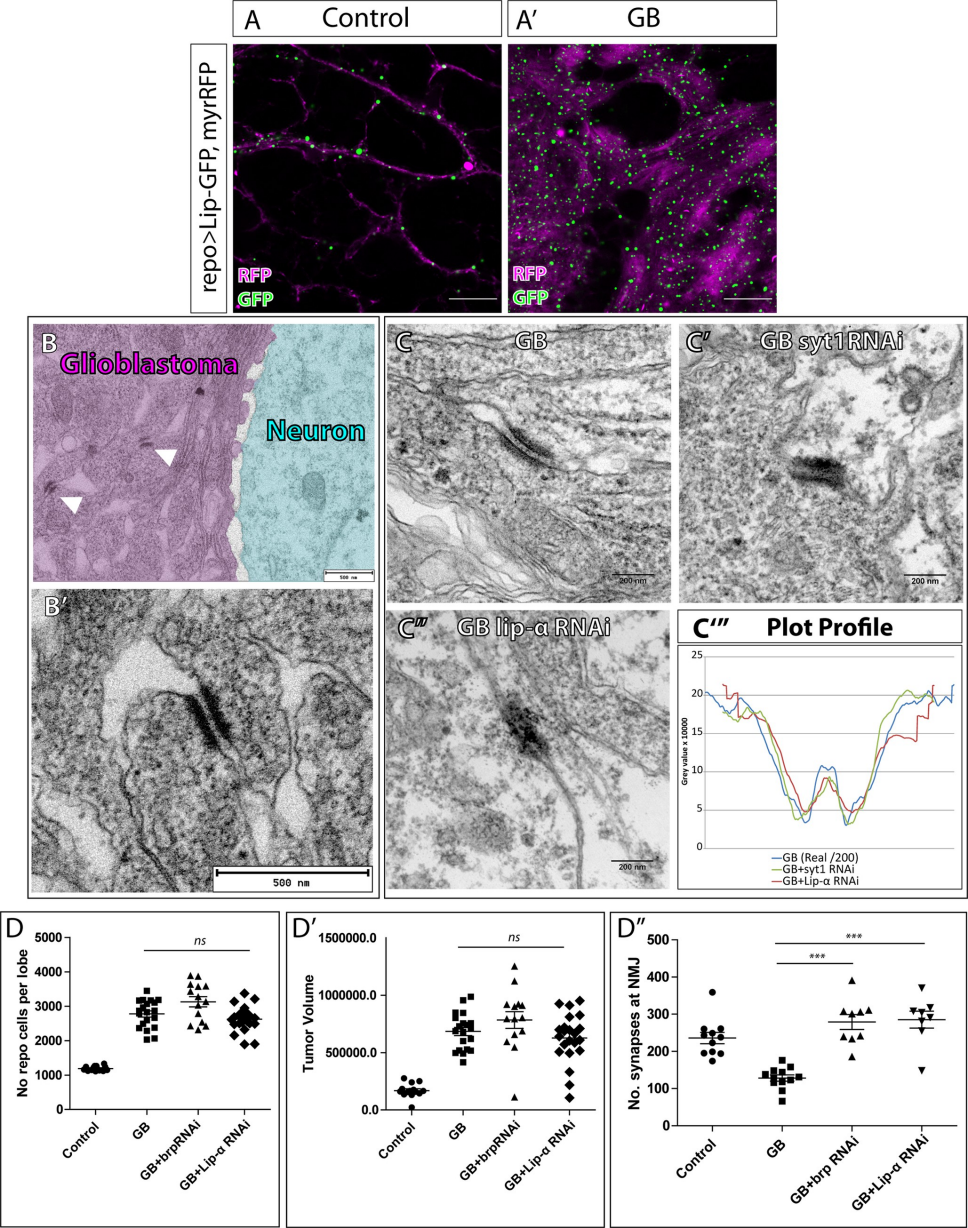
GB intratumoral synapses. A) Representative confocal images of Lip-α-GFP accumulation in glial cells (A) and GB cells (A’). Lip-α-GFP is in green and GB membrane in magenta. B) Representative TEM images of GB samples where glial membranes are labeled with HRP. B) GB is colored in magenta and neurons are in cyan. White arrowheads indicate electron dense signals between GB named “Intratumoral synapses”. B’) TEM Magnification of the intratumoral synapses. C) Comparison of Intratumoral synapses visualized with HRP in GB (C) and GB + *Syt 1 RNAi* (C’) or *lip α RNAi* (C”) expression, and plot profile of electron dense structures gray value in each genotype (C”‘. No.≥ 4 “intratumoral synapses”). GB control images are darker (x200) than experimental images (i.e. GB+Syt1RNAi or GB+lipRNAi). To compare control with experimental graphs we divided each GB point by 200. D) Quantification of glioma cells number (D), tumor volume (D’) or synapses number at the NMJ (D”) in control, GB, GB with *Brp RNAi* expression or GB with lip-α RNAi expression samples. No.≥ 8 NMJs. Statistics: One-way ANOVA (*ns* = non-significant) (D-D”) and Bonferroni’s Multiple Comparison Test (***p<0.0001) (D”). No.≥ 9 brain lobes. Scale bar: 15 μm (A-A’), 500 nm (B-B’), 200 nm (C-C”). Raw numbers and complete genotypes are in S1 Table.

The results obtained so far indicate the presence of presynaptic proteins in GB cells. Moreover, the presynaptic nature of GB cells has been demonstrated by the requirement of presynaptic proteins to calcium signal enhancement. Does that imply synaptogenesis between GB cells? We found Syt 1 accumulation in GB compared to controls (Fig 3A and 3B) concomitant with the lethality rescue observed upon s*yt 1* downregulation in GB (Fig 1A). *lip-α* downregulation in GB cells also rescues lethality (Fig 1A), as well as calcium enhancement (Fig 4A), besides, to further confirm the presence of Lip-α in GB we quantified Lip-α accumulation in normal glia and GB cells using a Lip-GFP reporter line. The data show accumulation of Lip-α -GFP in dots at the membrane of glial cells, and this accumulation increases in GB samples (Fig 5A and 5A’). This result shows that the localization and accumulation of the pre-synaptic protein Lip-α is enhanced in GB cells, compatible with the presynaptic nature of GB cells and the formation of synapses. By contrast, however, GRASP experiments had suggested that GB cells only function as post-synaptic structures with respect to neurons (see above, Fig 1B).

To clarify if pre-synaptic proteins indeed have a role in GB progression, we explored the possibility of synapses formation within the tumoral mass between GB cells, here defined as “intratumoral synapses”. To that end, we marked GB membranes with HRP and obtained transmission electron microscopy (TEM) images of GB samples and controls. EM images show high density structures between GB cells that are compatible with synapses (Fig 5B and 5B’). To validate if these densities exhibit synaptic features, we knocked down s*yt 1* or *lip-α* in GB cells and analyzed the tissue under TEM. The images show a morphological disruption of these electron densities upon *syt 1* knockdown (Fig 5C and 5C’) as well as upon *lip*-*α* knockdown (Fig 5C and 5C”). To measure the disruption of synaptic morphology by plotting the profile of TEM dense structures, we aligned all densities per genotype and plotted them, we obtained a graph that represents the mean of gray intensity in each point. GB control images are darker (x200) than experimental images (i.e. GB+*Syt1RNAi* or GB+*lipRNAi*). To compare control with experimental graphs we divided each GB point by 200. Control line showed that GB densities present a plateau with two peaks corresponding to a well-structured density bar. However, experimental samples showed a single peak where no bar is visible (Fig 5C”‘). These results suggest that the pre-synaptic proteins Syt 1 and Lip-α are functionally required to build putative GB-GB Intratumoral synapses.

Next, we analyzed the contribution of *brp* and *lip-α* pre-synaptic genes to GB progression. We quantified the number of GB cells and the volume of the tumoral mass upon *brp* or *lip*-*α* knockdown selectively in GB cells. The quantification of confocal images shows that *brp* or *lip-α RNAi* do not reduce the number of GB cells, nor the volume of the tumor (Fig 5D and 5D’). Nevertheless, our screen results indicate that Lip-α is required for GB causing lethality (Fig 1A), besides TMs analysis indicate that downregulation of *brp* or *lip-α* reduce the volume of TMs (Fig 3G), thus, we investigate the requirement of Brp and Lip-α for GB-induced synapse number reduction. We quantified the number of synapses at the NMJ in GB larvae and GB knocking down *lip-α* or *brp* specifically in GB cells. The results show that downregulation of *brp* or *lip-α* rescues synapse number at NMJ to normal values (Fig 5D”) and therefore prevents the reduction of synapse number in neurons caused by GB.

### JNK pathway activation depends on presynaptic gene expression

**Fig 6:**
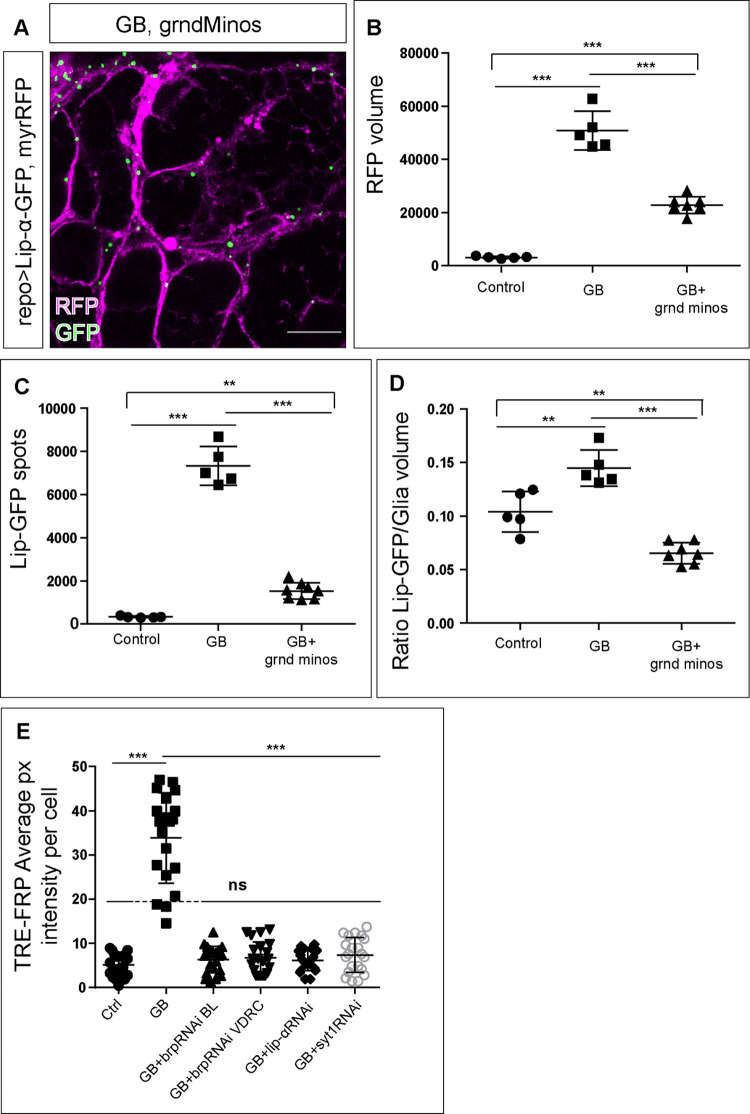
Presynaptic proteins are necessary for JNK upregulation in GB. A) Representative confocal images of Lip-α-GFP accumulation in the membranes of GB cells that express a dominant negative form of Grnd, *grnd Minos*. Lip-GFP is shown in green and GB membrane is shown in magenta. B) Quantification of glial membrane marked by mRFP in control brains, GB, and GB with *grnd minos* expressed in glia. No.≥ 5 animals. C) Quantification of Lip-α-GFP spots in control brains, GB, and GB with *grnd minos* expressed in glia. No.≥ 5 animals. D) Quantification of Lip-α-GFP signal normalized with glial membrane in control brains, GB, and GB with *grnd minos* expressed in glia. No.≥ 5 animals. Statistics in B-D: Bonferroni’s Multiple Comparison Test (**p<0.001; ***p<0.0001) E) Quantification of TRE-FRP signal intensity per cell in control, GB, GB that expresses *Brp lip-α* or *syt 1 RNAi*. Statistics: Dunnett’s Multiple Comparison Test (***p<0.0001; *ns* = not significant). No.≥ 5 images from 5 brain lobes. Scale bar: 15 μm. Raw numbers and complete genotypes are in S1 Table.

We have previously described that JNK activation is required for GB progression [5] so we wondered if this pathway was also related to the synaptic components. To answer this question, we overexpressed the dominant negative form of the JNK receptor Grindewall (*Grnd Minos*) [61] in GB samples. Upon downregulation of the JNK pathway, Lip-α-GFP accumulation and tumor volume were reduced compared with GB samples (Figs 5A, 5A’, 6A and 6B). Moreover, we quantified the total of lip-GFP spots as well as the ratio of Lip-GFP spots related to glial membrane and found that downregulation of JNK pathway reduces Lip-α accumulation in GB cells (Fig 6C and 6D). Finally we analyzed JNK pathway activation with the TRE-RFP reporter [62] in control brains, GB and GB with presynaptic genes (*brp*, *syt 1* or *lip-α*) downregulated (Fig 6E). The results indicate that the JNK pathway is upregulated in GB in line with our previous results [5,16]. In addition, downregulation of *brp*, *syt 1* or *lip-α* on GB rescue JNK activation to control levels.

### Synaptic genes rescue premature death caused by GB

**Fig 7:**
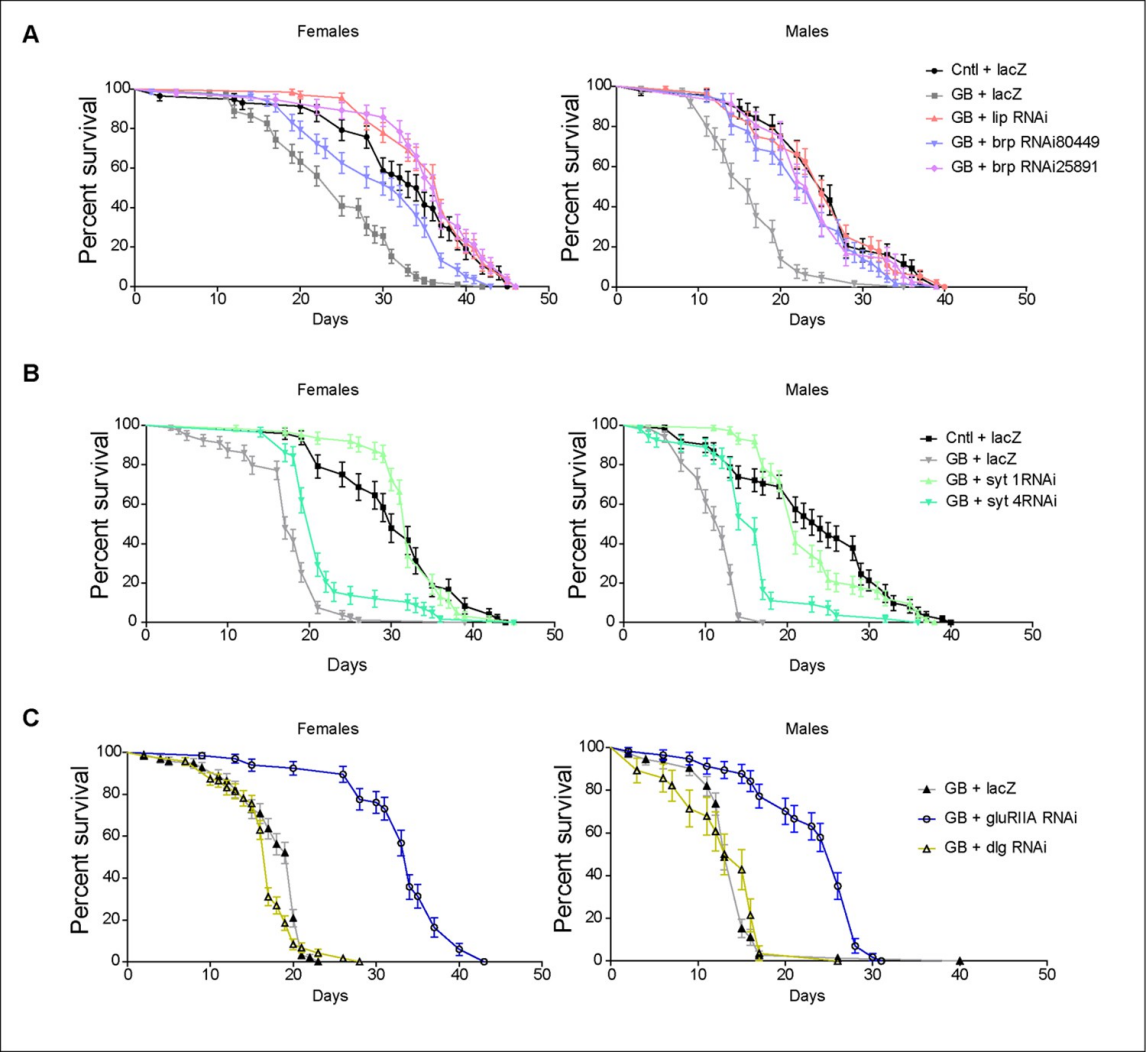
Synaptic genes contribute to premature death caused by glioblastoma. A) Graphs showing the percentage of survival of adult flies in control (black), GB (grey), GB + *lip α RNAi* (pale orange) and GB + *Brp RNAi* with two different RNAi lines (blue and purple). Females (left) and males (right) were analyzed separately. B) Graphs show the percentage of survival of control flies (black), GB (grey), GB + *Syt 1 RNAi* (bright green) and GB + *Syt 4 RNAi* (green). Females (left) and males (right) were analyzed separately. C) Graphs showing the percentage of survival of GB flies (grey), GB + *glu RIIA RNAi* (blue) and GB + *dlg RNAi* (yellow). Females (left) and males (right) were analyzed separately. No.≥ 30 animals. Logrank Test (Mantel-Cox) for trend analysis (All curve comparisons, ***p>0.0001). Raw numbers and complete genotypes are in S1 Table.

Finally, to determine the systemic impact of synaptic genes in GB, we evaluated the contribution of synaptic genes to the premature death caused by GB progression [5,6]. We measured the life span of adult male and female adult flies with GB, and compared it with flies in which certain pre- or post-synaptic genes had been knocked-down selectively in GB cells. The survival curves show in all cases that the induction of GB reduces the survival of male and female adult flies (Fig 7, grey lines). However, the knockdown of *brp* or *lip*-*α* prevents GB lethality and fully restores life span to control levels in males and females (Fig 7A). Also, the knockdown of s*yt 1*prevents GB-induced lethality (Fig 7B) suggesting that the expression of these pre-synaptic genes is required in GB cells to cause premature death.

In addition, we analyzed the contribution of *GluRII*, *dlg* and *syt 4* to life span in GB. The results show that *GluRIIA* or *syt 4* knockdown in GB cells, expands the life span of animals with GB, but the expression of *dlg* RNAi does not modify the premature death caused by GB progression (Fig 7B and 7C).

## Discussion

In addressing the mechanisms that facilitate cell to cell communication in GB progression, we found genes that encode for synaptic proteins and are required for GB progression, suggesting a contribution of synapses in GB. Previous reports using mice xenografts GB models indicated the existence of glutamatergic synapses structured as neuron pre-synaptic, and GB cells post-synaptic [19,20]. We have recapitulated neuron to GB synapses in *Drosophila melanogaster* and confirmed this unidirectional structure of glutamatergic synapses by GRASP experiments. We determined the expression and accumulation of post-synaptic proteins such as GluRII in GB membranes, in the close area of Brp pre-synaptic protein. Moreover, the expression of post-synaptic genes including *GluRII*, *dlg and syt 4* in GB cells is required for tumor progression, and for the deleterious consequences caused by GB, including neuronal synapse number reduction and premature death.

However, *dlg* knockdown in GB cells shows a particular phenotype, *dlg RNAi* prevents brain volume expansion and GB cells number increase, and also attenuates the synapse loss caused by GB but it is not sufficient to prevent premature death caused in GB. It is tempting to discuss the contribution of Dlg to GB progression, and furthermore, the requirements of Dlg in glial cells for the normal function. Dlg protein is involved in post-synaptic structures, but also in cell polarity, neuronal differentiation and organization, and septate junctions in cellular growth control during larval development. In addition, Dlg contains a guanylate kinase domain that suggests a role in cell adhesion and signal transduction to control cell proliferation [20,27,61–64]. In consequence, *dlg* knockdown could affect a number of cellular functions that reduces life span, independently of the prevention of GB progression.

The multiple functions of many proteins have recently emerged as a novel point of view in biology, and reconciles the complex mechanisms behind cellular physiology, and the limited number of known genes. For example, Troponin-I was described as a central player in muscle formation, but recent discoveries show that Troponin-I is also involved in apico-basal polarity, chromosome stability and tumorigenesis [65–67]. Moreover, Caspases are another example of multi-functional proteins involved not only in apoptosis, but also in cancer progression and quiescence [68–70]. Therefore, the impact of Dlg in GB and viability goes in line with this multiple functions of proteins, and brings a complex scenario with multiple functions for one single protein. These possible different functions for a protein depending on the cell type, or the specific status of the cells, is emerging as a robust hypothesis that might explain the presence of synaptic proteins in epithelial cells, the relation of muscle-associated genes to oncogenic proliferation, or the multiple roles of caspases in non apoptotic cells.

GluRIIA downregulation in GB reduces the tumor volume but not the number of cells. It has been demonstrated that GluR is required for proper cytoneme formation [44] and results from this work suggest that cytonemes and TMs share molecular mechanisms. Thus, we conclude that GluR is required for expansion and volume growth (through cytoneme/TMs) but not for cell number increase. Although we observed a reduction in GB cell number when combined with GluR mutant alleles we cannot ensure that this phenotype is caused by the lack of GluR in glial cells rather than caused by the lack of GluR anywhere else.

**Fig 8:**
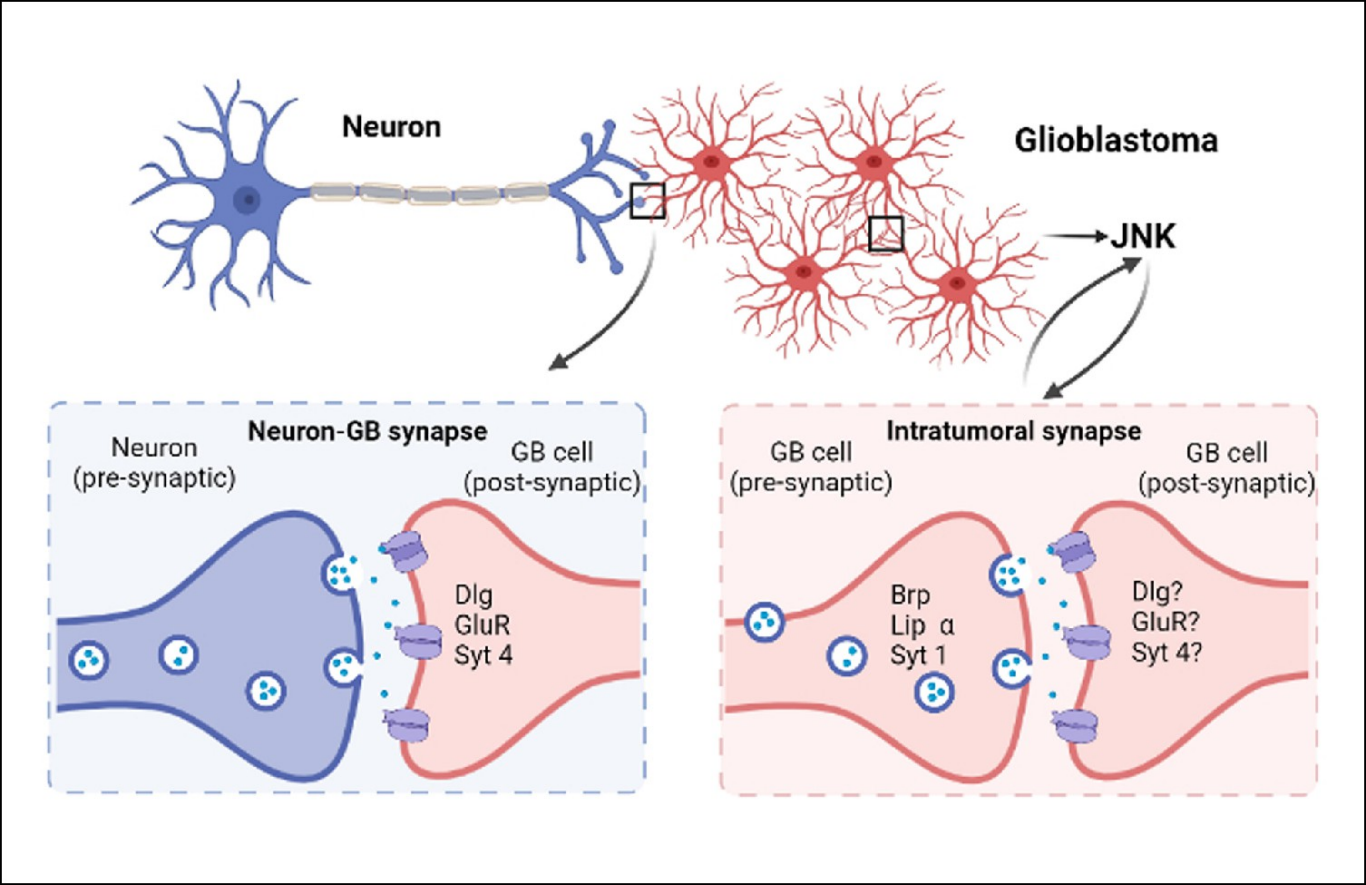
Summary. Schematic representation of neuron-GB synapse and intratumoral synapses. Bottom left, Neuron (blue) is the presynaptic component and GB cell (red) is the postsynaptic component. GB cells express postsynaptic genes *dlg*, *GluR* and *Syt 4*. Bottom right, intratumoral synapses are formed between two GB cells (red), presynaptic GB cells express *Brp*, *Lip α* and *Syt 1*. The other GB cells behave as postsynaptic, and have the same identity as in Neuron-GB synapses (*dlg*, *GluR* and *Syt 4)*.

Recent publications on the molecular mechanisms underlying GB progression have brought new information on the contribution of specific pathways including WNT, Neurologin, MMPs, Integrins, FAKs and JNK among others [5,16,18,71]. In particular, JNK pathway is involved in GB progression, and in the pro-synaptogenic pathway [38,72]. Therefore, the JNK pathway is of special interest in the study of GB and the contribution of synaptic proteins. We have analyzed the JNK pathway in this context and the results indicate that JNK pathway activation in GB cells is dependent on the expression of *brp*, *lip-α* and *syt 1*. Moreover, we used a dominant negative form of the receptor grnd to reduce the activation of the JNK pathway in GB cells, and the number of Liprin-GFP positive dots in GB cells was significantly reduced. In conclusion, these results suggest that the JNK pathway and the formation of synaptic structures among GB cells are mutually regulated. This molecular mechanism integrates in the signaling network that modulates the progression of GB cells, in relation with the GB-neuron cross communication (Fig 8).

### Pre-synaptic genes are required for GB progression

In addition, we investigated the contribution of synaptic genes that encode for pre-synaptic proteins, such as Lip-α, Syt 1 and Brp. Lip-α and Syt 1 appeared as hits in the unbiased genetic screening that we performed to search for anti-GB strategies, suggesting a contribution for pre-synaptic genes in GB. However, GB does not form any pre-synaptic structure with regards to the synapses established with neurons according to our GRASP results. Therefore, we discarded that the pre-synaptic components in GB are related to GB-neuron synapses. In consequence, we propose for the first time that GB cells establish intratumoral synapses and these are required for GB aggressiveness.

In particular, we have evaluated the contribution of Syt 1, Lip-α and Brp pre-synaptic proteins. We found that all of them contribute to calcium signaling enhancement in GB. Additionally, we demonstrated that GB cells accumulate Syt 1, determined by the signal of a Syt1-GFP fusion protein in GB tissue. The expression of a tagged Lip-GFP form in GB cells shows an increase in the dotted signal compatible with the formation of presynaptic structures in GB cells. These pieces of evidence support the formation of intratumoral synapses. We also obtained high resolution TEM images that show dense structures between GB cells, comparable to synaptic densities. These structures are morphologically disrupted upon *syt 1* or *lip-α* knockdown in GB, supporting the hypothesis of GB-GB synapses. Although *brp* or *lip-α* RNAi do not prevent GB cells number increase nor tumor volume increase, they do prevent synapse loss in neurons and life span shortening, features that correlate with GB progression. Altogether these evidences support the presence and contribution of intratumoral synapses to GB progression and the associated deleterious effects.

In spite of all these results, we cannot conclude that intratumoral synapses function as canonical bona fide glutamatergic synapses described in the nervous system. The results indicate that expression of synaptic genes in GB cells is relevant for the increase of intracellular calcium levels, which are associated with activity in glial cells GB progression and its negative consequences. In addition, we have observed the accumulation of synaptic proteins in GB cells and the formation of electron dense structures comparable to functional synapses.

Moreover, we have addressed the contribution of synaptic function in GB by gene expression knockdown of well-known key genes related to endocytosis, ionic channels or structural components of electric synapses. *Shibirie (Shi)* encodes the Dynamin, and is directly associated with endocytosis [73,74]. The effect of the temperature sensitive form of *Shibire (ShiTS)* in GB cell number increase is significant and is compatible with a contribution of vesicle recycling and synaptic activity to GB progression. We have previously described that GB cells increased the number of recycling endosomes, and that vesicular transport disruption by downregulation of *Kish*/*TMEM167*, involved in vesicular transport, disrupts EGFR turnover and therefore, limits GB growth [6]. Thus, we cannot discard that the contribution of Shi TS could be associated with other processes that require endocytosis during GB progression.

In addition, we have analyzed GB progression upon disruption of different genes involved in neuronal activity including Kir2.1 (human *KCNJ2*), *Shaker* (*KCNA1*), *KCNQ1*, *Ca beta1 (*human *CACNB4)*, *Achr alfa1* (*CHRNB2) and AchR alfa4* (*CHRNA4*). We also analyzed the contribution to GB progression of Shaking B, a structural component of the gap junctions at electrical synapses, and the expression of *tetanus toxin* (TNT) in GB cells to block synaptic transmission. The results indicate that *Shaker* and *Ca beta 1* GB cell number increase, suggesting a pivotal contribution in GB development. *Shaker* (Sh) encodes the structural alpha subunit of a voltage-gated potassium channel. It plays a key role in maintaining electrical excitability in neurons and muscle cells, as well as regulating neurotransmitter release at the synapse. Moreover, Sh interacts with dlg and therefore, we cannot reach a clear conclusion on the contribution of Sh to the pre- or post-synaptic identity of GB cells. Besides, *KCNQ1 is a potassium voltage-gated channel relevant for potassium transport*. *KCNQ1* RNAi did not show a significant effect on GB progression therefore, we cannot conclude a general contribution of potassium-dependent activity in GB progression.

On the other hand, *Ca beta 1 RNAi* expression in GB cells prevents GB progression and suggests that instead of a general electrical activity, calcium contribution might be of special relevance for GB progression. This conclusion is also supported by our results related to intracellular calcium levels. In conclusion, the majority of genes that participate in neuronal activity do not play an essential role in the increase of glial cells number in GB, suggesting that activity as a general feature is not relevant for GB progression, but the contribution of calcium and potassium signaling requires further study to determine the specific channels, mechanisms and relevance in GB cells.

### The relevance of synaptic genes in cancer

It is proposed that synaptic proteins have ancestral functions related to secretion of neurotransmitters in chonoflagellates, and conserved through metazoans. Moreover, studies on postsynaptic proteins in choanoflagellates revealed unexpected localization patterns and new binding partners, both which are conserved in metazoans [75,76]. New alternative functions of synaptic structures or synaptic proteins are possible and might not imply the transmission of impulses between two cells. There is evidence that indicates a coordination among glial cells under normal conditions, and in GB [46]. We have observed a reduction in the calcium influx in GB cells upon presynaptic and postsynaptic genes knockdown which suggests a functional contribution for synaptic genes to calcium influx in GB cells.

Downregulation of both pre and postsynaptic genes in GB reduces tumor volume and increases lifespan, thus, the expression of synaptic genes is required for GB progression. One possibility is that GB uses synaptic proteins to create a “neurosecretory apparatus” to interact with surrounding cells similar to what happens in choanoflagellates [75]. We have previously described that GB cells “vampirize” neurons, GB cells display a network of tumor microtubes that enwrap neurons and deplete Wingless/WNT from them. This imbalance in Wingless/WNT signaling promotes GB growth, causes a reduction in synapse number and premature death [5]. In other tissues, such as epithelial cells in *Drosophila* wing disc, cells express components of synapses that function in the formation of epithelial cytonemes and signaling [44]. This “neurosecretory apparatus” could be a mechanism to “vampirize” or exchange molecules with neurons, or with other GB cells.

Moreover, GB progression relies on the formation of cytoneme-like structures, tumor microtubes [5,14,45]. Cytonemes in epithelial cells accumulate receptors that contribute to cell-to-cell communication. In particular, components of neuronal synapses function in proper cytoneme formation and signaling including GluR, Syt 4 and Syt 1 [44]. Thus it is possible that by downregulating synaptic genes we were disrupting cytoneme formation and hence preventing GB growth.

Additionally, we propose that GB intratumoral synapses share functional features with *bona fide* mature synapses. Calcium signaling, TNT expression and defective synaptic vesicle trafficking in GB cells, rescues tumor volume as well as GB cells number. These results suggest that normal function of synaptic components in GB cells is necessary for GB growth. However, this synaptic functionality in GB cells might not be a neomorphic feature, rather than an imbalance of normal mechanisms used by glial cells. In our experience, neoplastic glia does not produce novel mechanisms, it rather uses already existing mechanisms that participate during development to grow, as it happened with Wnt or Impl2. Thus, we do not propose a neomorphic synaptic functionality rather than a specific requirement for GB cells to expand that depends on the contribution of *brp*, *syt1*, *lip-α* or *Caβ1* genes, and require the normal function of dynamin (Shi) and the endocytic vesicle system. Therefore, the precise mechanisms that underlie intratumoral synapses function in GB will have to be determined in the future.

Moreover, our findings go in line with the growing evidence indicating the involvement of synaptic proteins in the progression of GB but also in other tumors: [18] demonstrated that primary glioma cells express a repertoire of synaptic genes and form neuro-glioma synapses. An integrated bioinformatics analysis including 57 GBM cases and 22 cases of normal brain tissue found the Brp human orthologue ERC2 upregulated in tumor samples [77]. Besides, mutations in ERC2-RAF1 fusion have been detected in ganglio-gliomas [78], being RAF1 involved in MAP kinase pathway activation [79].

Likewise, other bioinformatics analysis identified SYT4 (human orthologue of Syt 4) as a potential core gene for glioblastoma. In this work the authors also identified other genes related to synapses such Synaptic Vesicle Glycoprotein 2B (SV2B) whose expression levels correlate with survival of patients [80]. Finally, according to the Human protein Atlas (www.proteinatlas.org/) the human orthologue of Lip-α, PPFIA1, is upregulated in a number of cancers including GB in which it has an eightfold increase in expression. In this database we also found that Dlg orthologues DLG1, DLG3 and DLG4 are overexpressed in many tumors including GB. Expression data indicate a 5.6, 2.5, and 12.5-fold increase of DLG1, DLG3 and DLG4 respectively. Besides, DLG3 and DLG4 high expression correlates with poor prognosis. Additionally, breast to brain metastasis is driven by activation of N-methil-D-aspartate receptors (NMDAR) through glutamate ligands. Metastatic tumor cells do not produce sufficient glutamate ligands to induce signaling, which is achieved by the formation of tripartite synapses between cancer cells and neurons [81]. Another example of enriched synaptic proteins in patients is found in gastrointestinal stromal tumors [82], thus it is plausible to hypothesize that synaptic gene overexpression could be a common mechanism for cancer progression.

Taking all these results together, GB progression depends on the expression of synaptic genes, in particular, the expansion of GB volume requires the expression of presynaptic and postsynaptic genes. In the case of *GluRIIA*, the number of GB cells is not reduced in GB upon *GluRIIA* knockdown, but knocking down all the other postsynaptic genes affect both GB cell number and tumor volume expansion. Our previous results [3] showed that the expression or *miR-8* in GB cells, prevented the expansion of GB volume but remained intact the number of GB cells. This is another example of mechanisms regulating different characteristic features of GB progression.

In conclusion, the results presented in this manuscript open new avenues to understand the mechanisms that coordinate the communication between GB cells, and the relation with the surrounding healthy cells and other tumoral cells. The strategies developed by neuroscientists during the last decades to modulate synapse number or activity emerge as a promising possibility to modulate the progression of GB, and maybe other tumors of the nervous system. The potential of these novel targets to prevent GB expansion deserve further investigation in line with the relevance of synaptic coordination within the tumoral mass.

## Supporting information

S1 Fig A) Quantifications of GFP intensity signal corresponding to GluRIIA-GFP protein in control brains, and brains upon GluRIIA knockdown by RNAi in glia (repoGal4). No.≥ 7 brain lobes. Statistics: T-Test (*p<0.05). A’) Quantifications of GFP intensity signal corresponding to GluRIIA-GFP protein in GB brains, and GB brains upon GluRIIA knockdown by RNAi in glia (repoGal4). No. = 9 brain lobes. Statistics: T-Test (***p<0.001). A”) Representative images showing GluRIIA-GFP in GB brains and GB brains upon GluRIIA knockdown by RNAi. B) Quantification of number of synapses in the NMJ in wt controls, upon expression of the RNAi used to downregulate presynaptic genes in neurons (elav-Gal4) and upon expression the RNAi used to downregulate postsynaptic genes in muscle (C57-Gal4). No.≥ 6 NMJs. Statistics: Bonferroni’s Multiple Comparison Test (***p<0.001). C) Quantification of number of glial cells and glial membrane volume in GB control, GB heterozygotic (GluR -/+) and GB transheterozygous (GluR -/-) for GluR mutant allele *Df(2)clh4/Df(GluRIIa-GluRIIb-)*^*Δ22*^. No.≥ 8 brain lobes Statistics: Dunnett’s Multiple Comparison Test (***p<0.0001). D) Representative confocal images showing Cameleon Calcium reporter in glial cells in control (D) and GB brains (D’). D”) Quantification of Camelenon signals in control and GB brains. Each point in the graph corresponds to the average fluorescence of different regions No. ≥6 brain lobes. Statistics: T-Test (**p>0.005). Scale bar: 50 μm.
(TIF)

S1 Table Results of graphics. This table shows the total numbers of all graphics.
(XLSX)
