# Supplementary figures and images for: Synaptic components are required for glioblastoma progression in Drosophila
Source: PLoS Genet. 2022 Jul 25;18(7):e1010329. doi: 10.1371/journal.pgen.1010329 (PMC9352205; doi:10.1371/journal.pgen.1010329)

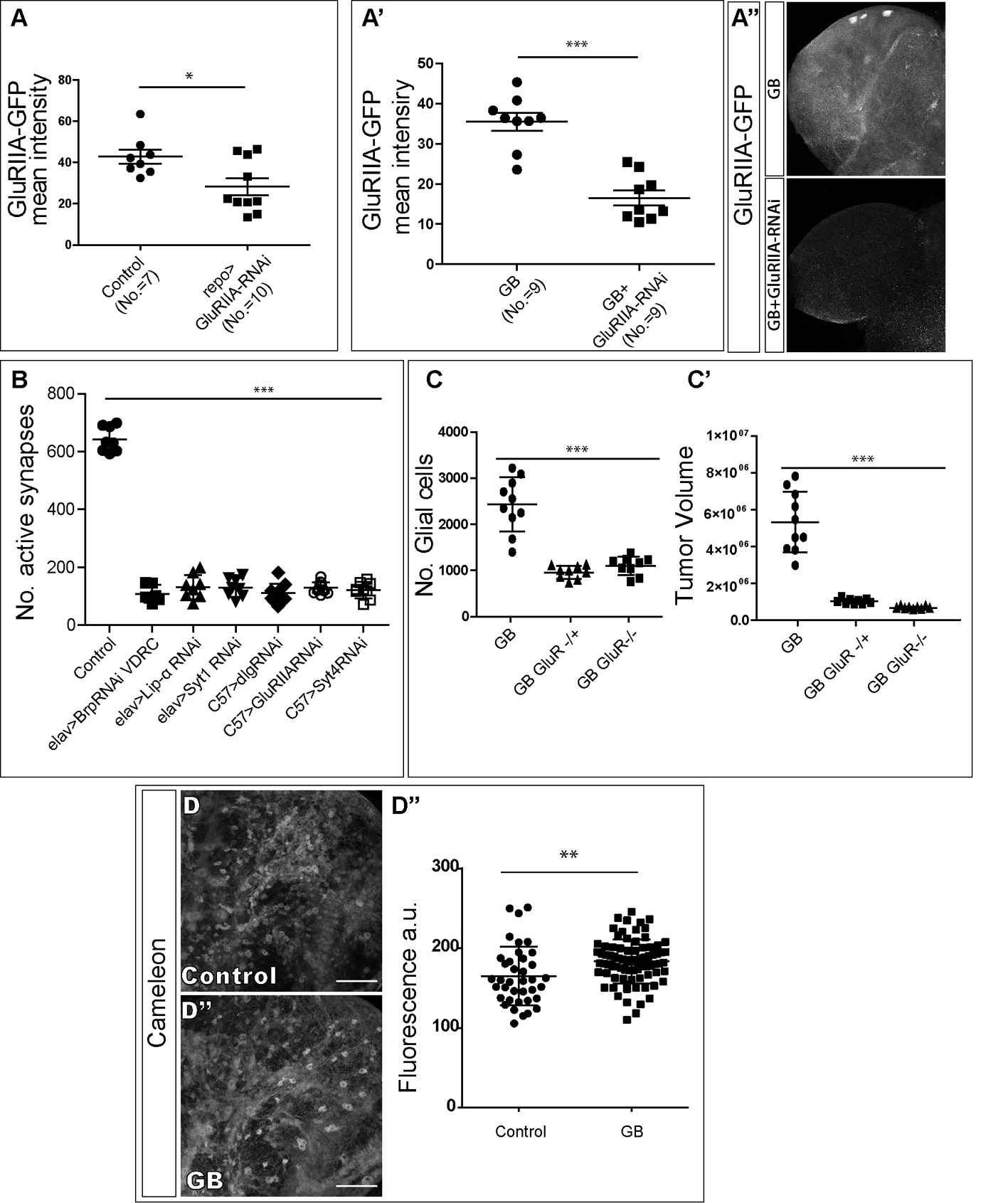

Supplement: S1 Fig — A) Quantifications of GFP intensity signal corresponding to GluRIIA-GFP protein in control brains, and brains upon GluRIIA knockdown by RNAi in glia (repoGal4). No.≥ 7 brain lobes. Statistics: T-Test (*p<0.05). A’) Quantifications of GFP intensity signal corresponding to GluRIIA-GFP protein in GB brains, and GB brains upon GluRIIA knockdown by RNAi in glia (repoGal4). No. = 9 brain lobes. Statistics: T-Test (***p<0.001). A”) Representative images showing GluRIIA-GFP in GB brains and GB brains upon GluRIIA knockdown by RNAi. B) Quantification of number of synapses in the NMJ in wt controls, upon expression of the RNAi used to downregulate presynaptic genes in neurons (elav-Gal4) and upon expression the RNAi used to downregulate postsynaptic genes in muscle (C57-Gal4). No.≥ 6 NMJs. Statistics: Bonferroni’s Multiple Comparison Test (***p<0.001). C) Quantification of number of glial cells and glial membrane volume in GB control, GB heterozygotic (GluR -/+) and GB transheterozygous (GluR -/-) for GluR mutant allele Df(2)clh4/Df(GluRIIa-GluRIIb-)Δ22. No.≥ 8 brain lobes Statistics: Dunnett’s Multiple Comparison Test (***p<0.0001). D) Representative confocal images showing Cameleon Calcium reporter in glial cells in control (D) and GB brains (D’). D”) Quantification of Camelenon signals in control and GB brains. Each point in the graph corresponds to the average fluorescence of different regions No. ≥6 brain lobes. Statistics: T-Test (**p>0.005). Scale bar: 50 μm. (TIF) [file pgen.1010329.s001.tif]
